# More Relaxation by Deep Breath on Methacholine- Than on Exercise-Induced bronchoconstriction during the Routine Testing of Asthmatic Children

**DOI:** 10.3389/fphys.2017.00768

**Published:** 2017-10-04

**Authors:** Iulia Ioan, Aurélie Tatopoulos, Stéphanie Metche, Laurianne Coutier, Emmanuelle Houriez, Sébastien Kiefer, Aurore Blondé, Claude Bonabel, François Marchal, Jocelyne Derelle, Cyril E. Schweitzer, Silvia Demoulin-Alexikova

**Affiliations:** ^1^Service d'explorations Fonctionnelles Pédiatriques, Hôpital d'enfants, CHRU de Nancy, Vandœuvre-lès-Nancy, France; ^2^EA 3450 DevAH, Faculté de Médecine, Université de Lorraine, Vandœuvre-lès-Nancy, France; ^3^Service de Pédiatrie, Hôpital d'enfants, CHRU de Nancy, Vandœuvre-lès-Nancy, France

**Keywords:** bronchial inflammation, airway mechanics, lung hysteresis, forced oscillations, childhood asthma

## Abstract

Deep inspiration (DI) dilates normal airway precontracted with methacholine. The fact that this effect is diminished or absent in asthma could be explained by the presence of bronchial inflammation. The hypothesis was tested that DI induces more relaxation in methacholine induced bronchoconstriction—solely determined by the smooth muscle contraction—than in exercise induced bronchoconstriction, which is contributed to by both smooth muscle contraction and airway wall inflammation. The respiratory conductance (Grs) response to DI was monitored in asthmatic children presenting a moderately positive airway response to challenge by methacholine (*n* = 36) or exercise (*n* = 37), and expressed as the post- to pre-DI Grs ratio (Grs_DI_). Both groups showed similar change in FEV_1_ after challenge and performed a DI of similar amplitude. Grs_DI_ however was significantly larger in methacholine than in exercise induced bronchoconstriction (*p* < 0.02). The bronchodilatory effect of DI is thus less during exercise- than methacholine-induced bronchoconstriction. The observation is consistent with airway wall inflammation—that characterizes exercise induced bronchoconstriction—rendering the airways less responsive to DI. More generally, it is surmised that less relief of bronchoconstriction by DI is to be expected during indirect than direct airway challenge. The current suggestion that airway smooth muscle constriction and airway wall inflammation may result in opposing effects on the bronchomotor action of DI opens important perspective to the routine testing of asthmatic children. New crossover research protocols comparing the mechanical consequences of the DI maneuver are warranted during direct and indirect bronchial challenges.

There has been a wealth of studies on bronchomotor tone regulation by the depth of breathing indicating that deep inspiration (DI) transiently reverses methacholine-induced bronchoconstriction in the otherwise healthy adult lung (Brusasco and Pellegrino, 1995; Pellegrino et al., 1998; Wang and Pare, 2003; An et al., 2007). Almost 20 years ago, DI has been suggested as a new tool to explore airway smooth muscle during lung function studies (Pellegrino et al., 1998). In contrast with the usual relaxation of the pretoned healthy airway smooth muscle, DI has more variable effect in asthmatic subjects, possibly reflecting the nature, degree and heterogeneity of inflammation and its impact on the airway wall (Crimi et al., 1992; Brusasco and Pellegrino, 1995) with potential practical implications for routine lung function testing (Pellegrino et al., 1998; Brown, 2014). Few studies have been performed in children. Comparing airway responses to methacholine and exercise has the potential to provide insights into underlying mechanisms of the effect of DI, because the airway reactivity to indirect challenges, e.g., exercise, is tightly linked to the presence of inflammation in the airway wall (Anderson, 2002), as opposed to direct activation of airway smooth muscle. The presence of inflammatory mediators that induce airway smooth muscle contraction may thus lead to different response to DI, compared to direct spasmogens that do not act through the release of inflammatory mediators. For about 10 years, our routine bronchial provocation protocols have been designed to standardize the airway response to DI. It was thought that retrospectively analysing such data would be of special interest to a better understanding of the mechanisms of these responses in different types of airway provocation. Here we review data from asthmatic children exhibiting a positive response to methacholine or exercise challenge. We hypothesized that, at a moderate level of bronchoconstriction, a DI would promote less reversibility when induced by exercise than by methacholine. The endpoint was the ratio of the post DI to pre DI respiratory conductance (Grs_DI_).

Children were referred to the lung function department by their pediatric pulmonologist. Asthma was defined by a complaint of wheezing, cough, dyspnoea or chest tightness at rest or on exercising and a positive response to methacholine or exercise challenge. The response to exercise was considered positive when FEV_1_ decreased at least 8% from baseline (Schweitzer et al., 2006). The response to methacholine was considered positive when FEV_1_ decreased at least 20% or Rrs increased at least 50% from baseline, at or below a cumulated dose of 1,200 μg (Marchal et al., 2002). Bronchodilator medications were discontinued at least 12 h prior to the testing and provocation allowed when the child had been free of respiratory symptoms for at least 2 weeks and baseline FEV_1_ was larger than 70% pred. Exercise and methacholine groups were matched for age, height, baseline FEV_1_ z-score, a moderate level of airway response to challenge and DI amplitude ranging 40–60% of the predicted FVC. Written informed consent was obtained from the children and their parents and the study was approved by the Ethics Committee (Comité de Protection des Personnes EST III, CHU de Nancy, Nancy, France). Procedures for spirometry, exhaled fraction of NO (FE_NO_), Grs_DI_ and challenges have been described previously (Marchal et al., 2002; Schweitzer et al., 2006). Acceptable Grs_DI_'s were analyzed at a 10–20% FEV_1_ decrease from baseline. Statistics were performed using Mann-Whitney, Chi square tests and multiple regression as required. Data are median and interquartile range.

Seventy-three asthmatic children (48 boys) were selected, 37 underwent exercise challenge and 36 methacholine (Table 1). There was no group difference in age, height, incidence of respiratory symptoms at rest, cough or wheezing on exertion, atopy or family history of asthma or atopy, rate of inhaled steroids prescription. In those tested for exercise however, there was a significantly higher incidence of exercise induced dyspnoea or chest tightness (*p* = 0.016), higher rate of prescription of montelukast (*p* = 0.05) and long-acting beta-agonists (LABA, *p* = 0.05). FEV_1_ z-score, FVC or FEV_1_ were similar (*p* ≥ 0.07) but FEV_1_/FVC was significantly lower (*p* = 0.0003) and FE_NO_ significantly higher (*p* = 0.016) in those tested for exercise. At the level of challenge examined, both groups showed similar FEV_1_ change from baseline (*p* = 0.06) and amplitude of DI (*p* = 0.35). Grs_DI_ did not differ between groups at baseline (*p* = 0.07), significantly increased after either challenge (*p* < 0.0001), and was significantly larger after methacholine than exercise (*p* = 0.014). The multiple regression analysis showed that Grs_DI_ was independent of FEV_1_ z-score, FEV_1_/FVC or FE_NO_ in either group, but correlated *positively* with the magnitude of FEV_1_ reduction after methacholine (*p* = 0.002) but not after exercise (*p* = 0.277).

**Table 1:** Characteristics of the children.

	**Exercise**	**Methacholine**	**p**
*n* (M/F)	37 (24/13)	36 (24/12)	
Age (year)	10 (8–11.6)	9.5 (8–11.5)	0.51
Height (m)	1.40 (1.30–1.50)	1.39 (1.27–1.51)	0.63
Cough	21/33	24/34	0.55
Wheezing	5/33	5/33	1.00
Dyspnea	5/33	9/33	0.23
Exercise induced cough	19/37	11/33	0.13
Exercise induced wheezing	8/37	4/33	0.29
Exercise induced dyspnea	23/37	11/33	*0.016*
Atopy	25/34	20/33	0.26
Family history of asthma	13/33	11/29	0.91
Family history of atopy	16/33	15/29	0.80
Prescription of montelukast	6/37	1/36	0.05
Prescription of LABA	6/37	1/36	*0.05*
Prescription of inhaled steroids	5/37	1/36	0.10
FE_NO_ (ppM)	31 (14–55)	16 (7–29)	*0.016*
FVC (L)	2.47 (1.96–2.83)	2.45 (1.87–3.03)	0.78
FEV_1_ (L)	2.09 (1.73–2.30)	2.17 (1.74–2.64)	0.58
FEV_1_ z-score	0.30 (−0.41–1.43)	0.81 (0.38–1.32)	0.07
FEV_1_/FVC	0.84 (0.81–0.92)	0.89 (0.88–0.93)	*0.0003*
ΔFEV_1_ test (%)	−13.4 (−16.3–11.4)	−15.6 (−17.4–12.6)	0.06
DI test (% FVCpred)	49 (42–54)	50 (46–54)	0.35
Grsi_DI_ at baseline	0.99 (0.90–1.1)	1.07 (0.96–1.15)	0.07
Grs_DI_ post-challenge	1.23 (0.96–1.5)	1.44 (1.21–1.7)	*0.014*

*LABA, long-acting beta-agonists; FE_NO_, exhaled fraction of NO; FVC, forced vital capacity; FEV_1_, forced expiratory volume in 1s; DI, deep inspiration amplitude; Grs_DI_, post DI to pre DI respiratory conductance. The italic values indicates the significant of difference*.

This assessment of several years practice of routine bronchial challenge in asthmatic children indicates that bronchodilation induced by DI, at comparable level of moderate airway obstruction, is of significantly larger magnitude when airways have been constricted by methacholine than exercise. The interpretative mechanical model is a central compartment representing the bronchioles and a peripheral one where more distal airways and lung tissue are lumped. Both compartments are coupled through the lung parenchyma attaching onto the intrapulmonary bronchiolar wall (Froeb and Mead, 1968). Energy dissipated on stretching the constricted bronchiolar smooth muscle explains bronchodilation after a deep breath (Brusasco and Pellegrino, 1995). This effect of DI was reported at baseline in a general population of school children (O'Connor et al., 2000), in school children with cough or asthma (Marchal et al., 2002), and in wheezy preschool children after methacholine (Milanese et al., 2000). In a small number of asthmatic adults, the effect of DI was similar whether airways were contracted by methacholine or hyperventilation of cold dry air, but the 40% change in lung resistance defining the bronchial responsiveness was perhaps insufficient to show a difference in response to DI between the tests (Malo et al., 1990). In fact, during indirect airway provocation, the bronchodilation by DI was demonstrated in asthmatic adults undergoing cold dry air challenge (Pichurko and Ingram, 1987; Lim et al., 1989) and in children with a history of wheezing after a free run (Schweitzer et al., 2006).

That airway inflammation impinges the airway response to DI has been shown when school children and teenagers with allergic asthma challenged with methacholine exhibiting poor bronchodilation by DI at sea level, readily bronchodilated to this maneuver after 3 months stay at altitude, a time during which resolution of airway inflammation was documented (Milanese et al., 2004). Similarly, adult asthmatics showed reversal of DI effect from bronchoconstriction to bronchodilation throughout the course of intensive therapy of acute asthma (Lim et al., 1989).

During exercise, two major consequences of inflammation are provoked by hyperventilation increasing water exchange between the respired gas and the airway. Mucosal dehydration and hyperosmolarity promote inflammatory mediator release with consequent airway smooth muscle constriction on the one hand, and engorgement in the bronchial circulation and oedema in the airway wall on the other (Anderson, 2002). The component of the airway obstruction related to fluid accumulation is unlikely to show similar resolution after DI compared with that related to smooth muscle constriction. Therefore, less bronchodilation by DI would be expected compared to the obstruction being entirely determined by the smooth muscle contraction. The prior report that DI-induced dilation of methacholine contracted but otherwise healthy airways is unchanged by the rapid saline accumulation in the airway wall (Pellegrino et al., 2003) may contradict such interpretation. However in that study, methacholine was administered at the same provocation dose both at control and saline infusion days, while the current study targeted similar degree of decrease in FEV_1_ with both exercise and methacholine. Therefore, the fluid shift to the airway during exercise induced bronchial obstruction may well have contributed to the smaller airway relaxation to DI compared with a similar degree of obstruction determined by methacholine. Moreover, with sustained hyperventilation, the gas-airway water exchange may extend into more distal airways, where smooth muscle contraction would increase hysteresivity of the peripheral component of the model described above, with the consequence that bronchodilation by DI would be attenuated or canceled (Brusasco and Pellegrino, 1995). The observation that the bronchodilation to DI is less during the late than the immediate response to allergen challenge may also be interpreted as resulting from inflammation extending into and contracting the terminal airways (Pellegrino et al., 1990).

Although bronchoprotection and bronchodilation by DI may be associated with different mechanisms, it is interesting to point that in asthmatic adults, the bronchoprotection by DI to methacholine challenge has been shown to be associated with low FE_NO_ and negative airway response to the indirect stimulus mannitol aerosol (Davis et al., 2014).

Due to the context of referral to the lung function laboratory and thus the lack of a randomized crossover design, the 2 groups showed slightly different phenotype. In those challenged with exercise, the more frequent use of LABA and montelukast, the higher FE_NO_ were consistent with more airway inflammation but the smaller FEV_1_/FVC was not associated with significant difference in Grs_DI_ at baseline. Earlier studies showed resolving severe airway obstruction was associated with reversal of the airway response to DI, from bronchoconstriction to bronchodilation (Lim et al., 1989). In contrast, in our study Grs_DI_ was *directly* related to the degree of induced airway constriction in that moderate range of acute methacholine-induced obstruction.

Interestingly, the airway wall exposure to inflammatory spasmogens has recently been shown to alter the force generating capacity of the airway smooth muscle that would become resistant to the stretching by DI (Bosse et al., 2011). Intrinsic alterations of the airway smooth muscle properties have been proposed to account for the variability of the response to DI in asthma. The role of airway smooth muscle hypertrophy has especially been identified as a factor limiting the ability of airway smooth muscle to stretch during simulated DI *in vitro* (Oliver et al., 2007). Altogether, it is possible that asthma history of different duration may impact such factors as the degree of airway smooth muscle hypertrophy and airway wall remodeling resulting, in the long term, in a different expression of DI between asthmatic children and adults.

Altogether the current data suggest the role of inflammation in attenuating the airway response to DI in children with exercise-, vs methacholine-induced bronchoconstriction. The findings present potential importance to lung function studies in children and deserve more systematic and exhaustive investigations, although research protocols involving crossover studies may be more difficult to accept by children. Other indirect airway provocation tests, e.g., mannitol or cold dry air hyperventilation, should be investigated for effect of DI. In addition, given the variety of asthma phenotypes, e.g., eosinophilic and neutrophilic (Hallstrand et al., 2005; Porsbjerg et al., 2009; Wang et al., 2011), it would be of interest to confront the response to DI to the precise nature of inflammation identified from sputum cellularity.

## Author contributions

II, FM, CB, CS, and SD have prepared the project of this study. II, SM, LC, SM, AT, CB, FM, CS, and SD managed preparatory phase of the study. AT, AB, JD, SK, EH, SM, CS, and LC performed participant recruitment. II, LC, SM, CB, FM, and SD performed lung function tests and assured technical assistance during challenge tests. II, SM, LC, CB, FM, CB, and SD performed data collection and statistics. II, AT, SK, JD, FM, CS, and SD have prepared the draft of manuscript. II, AT, LC, SM, FM, CS, and SD completed the work and revised the final manuscript.

### Conflict of interest statement

The authors declare that the research was conducted in the absence of any commercial or financial relationships that could be construed as a potential conflict of interest.
